# Radiation Therapy for Gestational Trophoblastic Neoplasia: Forward-Looking Lessons Learnt

**DOI:** 10.3390/cancers15194817

**Published:** 2023-09-30

**Authors:** Amelia Barcellini, Andrei Fodor, Alexandra Charalampopoulou, Chiara Cassani, Laura Deborah Locati, Raffaella Cioffi, Alice Bergamini, Sandro Pignata, Ester Orlandi, Giorgia Mangili

**Affiliations:** 1 Radiation Oncology Unit, Clinical Department, CNAO National Center for Oncological Hadrontherapy, 27100 Pavia, Italy; ester.orlandi@cnao.it; 2 Department of Internal Medicine and Medical Therapy, University of Pavia, 27100 Pavia, Italy; lauradeborah.locati@icsmaugeri.it; 3 Department of Radiation Oncology, IRCCS San Raffaele Scientific Institute, 20132 Milan, Italy; fodor.andrei@hsr.it; 4 Radiobiology Unit, Research and Development Department, CNAO National Center for Oncological Hadrontherapy, 27100 Pavia, Italy; alexandra.charalampopoulou@cnao.it; 5 Hadron Academy PhD Course, Istituto Universitario di STUDI Superiori (IUSS), 27100 Pavia, Italy; 6 Department of Clinical, Surgical, Diagnostic and Pediatric Sciences, University of Pavia, 27100 Pavia, Italy; ch.cassani@smatteo.pv.it; 7 Unit of Obstetrics and Gynecology, IRCCS, Fondazione Policlinico San Matteo, 27100 Pavia, Italy; 8 Translational Oncology Unit, Maugeri Clinical Research Institutes IRCCS, 27100 Pavia, Italy; 9 Unit of Gynaecology and Obstetrics, IRCCS San Raffaele Scientific Institute, 20132 Milan, Italy; cioffi.raffaella@hsr.it (R.C.); bergamini.alice@unisr.it (A.B.); mangili.giorgia@hsr.it (G.M.); 10 Department of Urology and Gynecology, Istituto Nazionale Tumori, IRCCS-Fondazione G. Pascale Napoli, 80131 Naples, Italy; s.pignata@istitutotumori.na.it

**Keywords:** gestational trophoblastic neoplasia, radiotherapy, radiation, rare cancer

## Abstract

**Simple Summary:**

The management of high-risk gestational trophoblastic neoplasia is multimodal, requiring a multimodality treatment that might include radiotherapy, especially in high-risk metastatic settings. The recent NCCN guidelines suggest considering whole-brain irradiation or stereotactic radiotherapy, with or without intrathecal methotrexate, for high-risk gestational trophoblastic neoplasia patients with brain metastases. However, there is little consensus regarding the indication and role of radiotherapy for the extra-central nervous system localizations such as lung, liver and vaginal. The haemorrhagic tendency justifies the haemostatic role of radiotherapy, but on the other hand, the high rate of necrosis is a radioresistance hallmark that requires great attention in terms of fractionation schedules and radiation type. Due to the rarity of this disease and the non-routine use of radiotherapy in metastatic settings prospective randomized trials are unrealistic. In this narrative review, we discuss the data about the past and current roles and indications of radiotherapy concerning these patients to potentially help physicians in treating these challenging clinical presentations.

**Abstract:**

Gestational trophoblastic neoplasia (GTN) includes several rare malignant diseases occurring after pregnancy: invasive moles, choriocarcinoma, placental site trophoblastic tumours, and epithelioid trophoblastic tumours. Multidisciplinary protocols including multi-agent chemotherapy, surgery, and occasionally radiotherapy achieve good outcomes for some high-risk metastatic patients. In this narrative review of the published studies on the topic, we have tried to identify the role of radiotherapy. The available studies are mainly small, old, and retrospective, with incomplete data regarding radiotherapy protocols delivering low doses (which can make this disease appear radioresistant in some cases despite high response rates with palliative doses) to wide fields (whole-brain, whole-liver, etc.), which can increase toxicity. Studies considering modern techniques are needed to overcome these limitations and determine the full potential of radiotherapy beyond its antihemorrhagic and palliative roles.

## 1. Introduction

Under the umbrella of gestational trophoblastic neoplasia (GTN), there is a spectrum of rare malignant diseases: invasive moles, choriocarcinoma, placental site trophoblastic tumours (PSTTs), and epithelioid trophoblastic tumours (ETTs) [1,2,3,4,5,6,7]. Since GTN can occur after any type of pregnancy, the true frequency of GTN is unknown, even though recent data suggest that choriocarcinoma occurs in 1:50,000 deliveries and ETTs represent 0.2% of all UK gestational trophoblastic diseases (including the premalignant forms) [8,9]. Extremely rare cases of GTN arising in non-pregnant and post-menopausal women (non-gestational choriocarcinoma) have been reported in the literature [1,10]. The most common onset symptom is abnormal vaginal bleeding followed by amenorrhea or district-specific symptomatology due to the presence of metastases (i.e., neurological alteration and localized pain, and/or bleeding) [1,10]. GTNs are scored according to a prognostic and anatomic staging system predicting the possible single-drug chemoresistance and segregating it into low-risk (scores between 0–6) and high-risk (≥7) [11]. Indeed, single agents can be administered to low-risk patients for whom a multi-agent drugs approach has been suggested in case of resistance to monotherapy. In addition to the lower possibility of sensitivity to a single-drug agent, high-risk GTN patients often experience distant metastases, mainly occurring in the lung (80%), followed by the vagina (30%), brain (10%), and liver (10%), with the localization of the last two characterized by the worst prognosis, particularly if co-occurring [12,13]. Among GTNs, placental site trophoblastic tumours and epithelioid trophoblastic tumours are characterized by slow growth, high-risk lymph node involvement, a lower production of hCG, and the tardive tendency to metastasize. Currently, managing high-risk GTN is often multimodal, as several cases require a combination of multi-drug chemotherapy and surgery [1]. The recent NCCN guidelines suggest considering whole-brain radiation therapy (WBRT) or stereotactic RT (SRT) with or without intrathecal methotrexate for high-risk GTN patients with brain metastases [5].

However, in these circumstances, there is little consensus regarding the indication and role of RT. All types of GTN originate from the normal placenta, representing the abnormal counterparts of the villous and extra-villous (interstitial) trophoblast. In particular, choriocarcinomas are epithelial hCG-secreting tumours characterized by a villous trophoblast phenotype, a substantial amount of haemorrhaging and central necrosis, and a biphasic architecture resembling cytotrophoblast-like and multinucleate, pleomorphic, syncytiotrophoblast-like structures [14]. The malignant opposing parts of the extra-villous interstitial-implantation-site-like trophoblasts are the placental site trophoblastic tumour and epithelioid trophoblastic tumour that develop less necrotic, haemorrhagic, uterine lesions with lower vascular invasion. If, on the one hand, the haemorrhagic tendency justifies the haemostatic role of RT, on the other hand, the high rate of necrosis is a radioresistance hallmark that requires great attention in terms of fractionation schedules and radiation type [15,16,17,18]. RT has been used in the past mainly in the treatment of brain and liver metastases of GTNs. However, thanks to the increased efficacy of systemic therapies and the advancements in supportive therapy (i.e., the use of granulocyte colony-stimulating factor to prevent neutropenic complications and treatment delays), RT indications are currently more restricted [2,5,19].

Given the rarity of this disease; the importance of tailored and often multimodal treatment, particularly in the case of chemoresistant disease; and the technical advances in RT techniques, we aimed to critically review the available literature. Our aim was to summarize the data about the past and current roles and indications of RT in relation to these patients to potentially help physicians in treating these challenging clinical presentations of GTNs.

## 2. Material and Methods

The design of the current manuscript followed the recommendations on the quality assessment of narrative review articles provided by Baethge et al. [20]. From November 2022 to March 2023, two authors (A.Ba. and A.F.) independently searched for articles reporting cases of GTNs treated with RT, incorporating the terms “radiotherapy” and “gestational trophoblastic neoplasia”, in the most significant medical databases (PubMed Cochrane Database of Systematic Reviews, EMBASE, and Web of Science). Only publications in English were considered. No limitations regarding publishing date or article type were applied. Duplicates were eliminated, and articles were collegially discussed. Then, a backward literature search for the selected article was made in order to identify additional papers by checking the references of the reviews in addition to references cited in the relevant articles already selected.

## 3. Results

### 3.1. RT for Brain Metastases of GTN

Due to their rarity, the data concerning GTNs with central nervous system (CNS) involvement derive from heterogeneous, retrospective, and small series. For these reasons, the GTNs’ real incidence and prevalence are unknown. In 1096 autoptic cases of solid tumours, Chason et al. identified 200 brain metastases (BM), of which only 1 (0.5%) was from choriocarcinoma [21]. No cases were found, however, in the 393 autopsies conducted by Hunter and colleagues [22] either in autoptic or retrospective studies [23,24,25]. In the review of the literature conducted by Piura et al. [26], GTN BMs were documented in 222 cases overall, with an estimated incidence of about 11% in living GTN-afflicted women. Only 11.3% of the cases featured the involvement of the CNS in the first systemic presentation of GTNs, which was generally (i.e., 90% of the cases) part of a multi-metastasis disease [26]. Multiple BMs or posterior fossa involvement are more likely to present with headache as the most common symptom (50% of cases); neurological deficits can be seen in up to 40% of cases, and seizures occur in 10–15% [27,28,29,30,31,32,33]. GTN BMs are rarely asymptomatic [34]. Even if the clinical and radiological manifestations of GTN do not differ from other BMs, GTN BMs present a major risk of haemorrhage [35,36,37]. BMs from GTNs are treated with a combination of cytotoxic chemotherapies, and this has significantly improved clinical outcomes in recent years. In particular, the most used protocols are the following: EMA-CO (Etoposide + Methotrexate + Actinomycin D + Cyclophosmamide + Vincristine + Folinic Acid); EMA-EP (Etoposide + Methotrexate + ActinomycinD + Etoposide + Cisplatin + Folinic Acid); FAEV (Floxuride + ActinomycinD + Etoposide + Vincristine); and Intratechal methotrexate with concomitant EMA-CO/EP [12]. The development of new BMs during systemic treatment is an indicator of chemoresistance and worse outcomes [36], and a multimodal treatment including surgery and RT may be an option. To the best of our knowledge, there have been 270 cases of BMs from GTNs treated with RT (Table 1). When the age at diagnosis was available, it ranged between 14 and 54 years. Between 1957 and 2019, 93.70% of cases (N = 253/270) underwent WBRT. WBRT was delivered mainly with Cobalt-60 RT or 4–6 MV parallel opposed fields of photon beam RT with a total dose ranging between 2 and 40 Gy (with 400–600 rad being disclosed in older reports, which is equivalent to 4–6 Gy). The data about RT toxicities for these older RT techniques were reported for only 62.2% (N = 168/270) of the irradiated women, namely, 66.4% (N = 168/253) of the WBRT cohort. No neurological sequelae were reported in 62 cases (24.5% of the WBRT cohort and 16% of the whole series) [38,39]. Severe proliferative retinopathy was reported in 39 cases (15.4% of the WBRT cohort and 14.4% of the whole series) [32]. One patient died after one fraction for cerebral herniation due to intracranial oedema [33]; one experienced a seizure after the successful completion of RT [40]; and there was one case of bilateral radiation-induced parotitis [41], one case of RT-otitis media [41], and one case of optic atrophy after WBRT [41]. The clinical outcomes after WBRT were inhomogeneously described or difficult to stratify in the series, and it was often unclear whether RT alone could eradicate the disease. Weed et al. [27] reported the survival of 14 GTN patients with BM, 13 of whom had been treated with WBRT and systemic chemotherapy, with 7 (50%) achieving a complete response. Seven of the thirteen patients (53.8%) treated with WBRT survived over a follow-up of 12–120 months [27]. In a retrospective analysis of 19 BMs deriving from GNT conducted by Jones et al. [39], five (26.3%) patients treated with a combination of methotrexate, actinomycin D, and chlorambucil and WBRT were alive, with an overall survival (OS) ranging between 3 days and 15 years. Among 42 patients who underwent multiagent chemotherapy with methotrexate, actinomycin D, and chlorambucil (MAC)- or etoposide-based regimens + WBRT, Evans et al. [32] reported an OS of 44%, which seemed to be influenced by hCG level, the size/number of metastases, and the timing of a BM’s onset (a worse OS was reported for the development of a BM during active therapy). Moreover, the control of extra-CNS disease seemed to significantly impact the OS [33]. Indeed, in the series reported by Schechter et al. [33], the 2- and 5-year actuarial survival of patients ranged between 100% and 83%, respectively, for patients with extra-CNS control of disease, and 8% and 0%, respectively, for those with no systemic control (*p* = 0.0002). Therefore, symptoms at presentation also affected survival, which ranged from 100% (8/8) for asymptomatic women to 41% (7/17) for symptomatic ones in the analysis by the Brewer Trophoblastic Disease Center [42]. The local control (LC) achieved via WBRT seemed promising. Brace et al. [38] reported no presence of disease upon autopsy in 5 of the 21 analysed patients. In the GNT BM cohort analysed by Jones et al. [39], 26.3% showed no evidence of disease from 4 to 15 years after the diagnosis of brain metastases. The total RT doses ≥ 22 Gy significantly impacted the 5-year actuarial LC [33]. In the same series, 7 of the 21 patients treated experienced a persistent or progressive disease at initial sites; 4 presented new BMs, and 10 patients were free from intracranial disease progression [33]. None of the 11 patients treated at the Prentice Women’s Hospital of Chicago had uncontrolled brain disease [43].

Overall, sixteen women (6.5%) between 1990 and 2020 were treated with SRT, and one was treated with Gamma Knife^®^ (Elekta AB, Stockholm, Sweden^)^, but there was a lack of technical RT details about these approaches (Table 1). Soper et al. [44] reported a 15-month remission for the only patients with high-risk GTN treated with Radionics XKnife™ (Burlington, MA), an RT LINAC-based radiosurgery system, for two BMs (a parieto-occipital BM with a total dose of 15 Gy at the 96% isodose line, TVR = 2.2; a frontal BM with a total dose of 18 Gy prescribed at the 95% isodose line, TVR = 3.5). The only patient treated in the series reported by Gavanier et al. [31] with SRT after metastasectomy had no BM at the time of analysis. A beta-hcG suppression was registered after Gamma Knife^®^ radiosurgery in one of the four irradiated cases analysed by Xiao et al. [45]. No details about brain toxicities were recorded.

**Table 1:** GTN-induced brain metastases treated with RT.

Author	Years of the Analysis	Pts with BM	Pts Irradiated for BM	Age (Range)	Type of RT	Details of RT	Dose RT	Toxicity	Intracranial Disease Control	Survival Outcomes
Brace, 1968 [38]	NA	21	21	NA	WBRT	2 Mev Van De Graaf machine; large fields	600–2000 rad (=6–20 Gy)	None of the survivors presented signs of toxicity	5/21 showed no tumours upon autopsy	5/21 survived
Weed, 1980 [27]	1966–1979	14	13	20 (15–39)	WBRT	Co60	2000–4000 rad (=20–40 Gy) for 10 days	One case of radionecrosis	2/13 patients needed a second course of 2000 rad (=20 Gy) irradiation for persistent CNS lesions 2 years and 4 months, respectively, after their first irradiation	7/13 patients survived (53.8%) for 12–120 months
Athanassiou et al., 1983 [28]	1957–1981	33	13	NA	WBRT	Co60	3000–4500 rad (=30–45 Gy)	NA	Stratification was not possible within the entire cohort	Authors reported: “we have no able to show that RT alone is capable of eradicating the disease”
Barnard et al., 1986 [46]	1966–1980	NA	6	Stratification was not possible within the entire cohort	WBRT	Co60	2000–4000 rad	NA	NA	NA
Yordan et al., 1987 [47]	NA	78	18	NA	WBRT	NA	NA	NA	NA	50% survived, but none of the deaths were due to central nervous system involvement.
Rustin et al., 1989 [48]	1957–1980	13	2	Stratification was not possible within the entire cohort	WBRT	NA	NA	NA	NA	Authors reported: “ its value is unclear due to the subsequent chemotherapy”
Jones et al., 1990 [39]	1967–1987	19	19	19–54	WBRT	Co60	1500–3000 rad	No neurological sequelae	5 pts NED (26.3%) at 4, 5, 8, 14, 15 years	OS 3 days–15 years
Evans et al., 1995 [32]	1966–1992	42	42	NA	WBRT	NA	30 Gy	39 cases of severe proliferative retinopathy; 2 pts with residual neurological deficits after remission; 1 pt with left hemiparesis and requiring a tracheostomy.	NA	OS = 44%
Ayhan et al. 1996 [35]	1978–1993	7	6	28.7 (20–34)	WBRT	NA	30 Gy	NA	NA	Mean survival of 6.8 months; overall mortality ratio of 85.7%; causes of mortality were intracranial haemorrhage in four, elevated intracranial pressure and herniation in one, and granulocytopenia and sepsis following chemotherapy in one patient
Small et al., 1996 [40]	1962–1994	26	26	14–43	WBRT	Cobalt-60 or 4-MV photons delivered in parallel opposed fields. The dose was prescribed to be delivered at midplane.	23.86–40 Gy	1 pt—seizures after successful completion of therapy	In the symptomatic patients (group A) who completed WBRT with concurrent chemotherapy, there was no evidence that cranial disease persisted	Overall 5-year actuarial survival rate = 51%
Schechter et al., 1998 [33]	1967–1994	21	21	35 (19–54) years	WBRT	Co60 or 6 MV Photons	22 Gy (2–36)	1 pt died after one fraction via cerebral herniation due to intracranial oedema; the neurological status of long-term survival was stable or improved compared to that upon BM diagnosis	7/21 persistent or progressive disease at initial sites; 4/21 presented new BM; 10/21 were free from intracranial disease progression. 5-year actuarial LC with > or = 2200 cGy was 91% vs. 24% with <2200 cGy (*p* = 0.05);	2- and 5-year actuarial survivals of the 9 patients whose disease was controlled at extracranial sites were 100 and 83%, respectively, vs. 8 and 0%, respectively, for the 12 whose extracranial disease was not controlled (*p* = 0.0002). Four (33%) patients had persistent or progressive extracranial disease and developed new sites of brain metastases, compared to 0% of the patients whose extracranial disease was controlled (P = 0.05) (*p* = 0.05)
Ghaemmaghami et al., 2004 [49]	1996–2001	9/40	9	30 (17–53) years	WBRT	NA	30 Gy in 10 consecutive days	No neurological sequelae	63% of patients responded to high-dose EMA-EP and concurrent WBRT	56% responded and 44% died
Soper JT et al. 2007 [44]	2003–2005	4	1	NA	SRT	Radionics XKnife™ RT LINAC-based radiosurgery system	Parieto-occipital BM 15 Gy at the 96% isodose line, TVR = 2.2; frontal BM with a total dose of 18 Gy prescribed at the 95% isodose line, TVR = 3.5	No neurological sequelae	15 months	Prolonged remission (15 months); patient experienced a neutropenic sepsis with respiratory distress syndrome
Neubauer et al., 2012 [43]	1962–2009	37	37	NA	WBRT	NA	24–40 Gy or 24–30 Gy	NA	None of these patients had uncontrolled brain disease upon death.	OS increased from 51% between 1962 and 1994 to 64% between 1995 and 2009, including for 3 (60%) of 5 patients who developed brain metastases during treatment
Alifrangis et al., 2013 [50]	1995–2010	17	7	Stratification was not possible within the entire cohort	SRT	Areas of residual intracranial metastases after chemotherapy	NA	NA	Stratification was not possible within the entire cohort	Stratification was not possible within the entire cohort
Savage et al., 2015 [51]	1991–2013	27	5	Stratification was not possible within the entire cohort	SRT	NA	NA	NA	Stratification was not possible within the entire cohort	Stratification was not possible within the entire cohort, but none of the five patients, treated with stereotactic radiotherapy for persistent brain lesions after chemotherapy, died
Xiao et al., 2015 [52]	1990–2013	109	2	Stratification was not possible within the entire cohort	SRT	NA	NA	NA	Stratification was not possible within the entire cohort	Stratification was not possible within the entire cohort
Gavanier et al., 2019 [31]	1999–2016	21	1	NA	SRT	After metastasectomy	NA	NA	NED	NED at the time of analysis
Xiao et al., 2022 [45]	2006–2020	14	4	21–48	3 WBRT, 1 Gamma Knife^®^ *		NA	NA	NA	negativisation of bhCG; 1 pt delivered a healthy baby
Gonzales-Acantilado et al. 2022 [41]	2010–2019	29	17	Stratification was not possible within the entire cohort	WBRT	NA	30 Gy	Bilateral radiation-induced parotitis (1/17); radiation-induced otitis media (1/17); optic atrophy (1/17)	NA	11/17 (64.70%) achieved biochemical remission.

WBRT = Whole-Brain Radiation Therapy, Co60 = Cobalt-60 teletherapy, SRT = Stereotactic Radiation Therapy, TVR = Treatment Volume Ratio (total volume receiving the prescription dose divided by the tumour lesion volume); * Gamma Knife^®^ (Elekta AB, Stockholm, Sweden).

### 3.2. RT for Extra-Central Nervous System Metastases of GTN

Between 1.8% and 7.7% [53,54] of GTN patients, and up to 19% of women with stage IV GTN, experienced liver metastases [55]. The survival rates were once quite poor (10–55%) but have improved in the last few years thanks to the introduction of FAEV and EMA/CO regimens [54,56,57]. The literature data regarding the use of RT for the treatment of liver GTN metastases are very scarce and limited to retrospective data concerning small and mono-institutional series. No indications about RT in this setting are provided by the relevant guidelines [1,5]. Indeed, to the best of our knowledge, only two studies reported the outcome of 18 of 27 (66.6%) patients with liver metastases treated with whole-liver RT [39,46]. These patients, with an age ranging between 19 and 54 years, were irradiated with Cobalt-60 RT with a total dose of 1400–2400 rad (=14–24 Gy) [39,46]. The lower total doses delivered were related to the treatment aim, which was mainly haemostatic or the avoidance of bleeding. Two of the seven cases analysed by Jones et al. [39] were re-treated with 3400 and 4400 rads (=34–44 Gy). Barnard et al. [46] reported the long-term outcomes of two of eleven women whose livers were irradiated, with an overall survival of 132 and 186 months. There is a lack of toxicity data, with the description of only one case of hepatotoxicity with undetailed serum enzyme elevation [46].

Up to 80% of patients presented lung involvement at the time of diagnosis [1,2]. Chemotherapy is the gold standard in this setting, but when there are histology-related doubts, surgery is recommended. There is a serious lack of data about the use of RT. Ngan et al. [58] suggested RT with a palliative aim when chemotherapy and surgery are not feasible.

Vaginal metastases are found in 10 to 36% of GTN cases [59]. They originate within the submucosal venous plexus of the vagina and are characterized by highly abnormal and fragile vessels [60]. The main symptoms are bleeding and purulent discharge. In these cases, packing or angiographic embolization can be combined with systemic therapy [61]. For cases with a risk of haemorrhage, biopsy or surgery are not recommended [36,59,60]. Ngan et al. [2] recommended palliative RT for patients unfit for the above-reported approaches.

## 4. Discussion

GTNs are a group of rare and frequently metastasizing tumours originating from the placenta characterized by improper trophoblast proliferation. The International Federation of Gynecology and Obstetrics (FIGO) prognostic score system divided GTNs into two groups (low- and high-risk) based on the risk of developing resistance to mono-chemotherapy with MTX or actinomycin-D [2].

For low-risk GTN (FIGO Stages I–III: score < 7), single-agent chemotherapy is the gold standard, although additional agents can be worthwhile in some cases, especially for scores of 5–6 that are associated with chemoresistance [2]. The treatment of high-risk GTN (FIGO Stages II–III: score ≥ 7 and Stage IV) is based on a multidisciplinary approach including multi-agent chemotherapy, surgery, and RT. Up to 80% of high-risk GTNs can lead to the development of distant metastases of the lung, 30% of the vagina, 10% of the brain, and 10% of the liver [12]. We have detailed the use of RT in the metastatic setting, especially for treating patients with BMs or liver metastases, to reduce haemorrhagic complications. The RT data were mainly derived from retrospective, mono-institutional, and small series; moreover, details concerning RT volumes, doses, and techniques/fields, important factors to be examined in the analysis of loco-regional control and toxicities, are lacking. Furthermore, another bias to consider is the long timespan covered by the analysed cohort, which is related to crucial changes in RT technologies, planning, and dose delivery. For these reasons, if RT is considered in cases of BMs, the current guidelines do not apply to the other localizations analysed in the current narrative review.

With regard to BM, when available, the total doses ranged from 2 to 40 Gy for WBRT and were often delivered concurrently with chemotherapy with haemostatic and tumouricidal aims, without detecting toxicity [62]. However, in these series, it was not possible to evaluate whether RT alone was able to control the disease and whether the wide range of doses was a bias. On the other hand, RT increases the permeability of the blood–brain barrier and could improve drug concentrations and, subsequently, chemotherapy results [63].

Concerning the toxicity after WBRT, data were reported only for 66.4% (N = 168/253) of patients, with thirty-nine cases (15.4% of the WBRT cohort and 14.4% of the entire series) of severe proliferative retinopathy [32], one case of intracranial oedema leading to death via cerebral herniation [33], one case of seizures [40], one case of bilateral radiation-induced parotitis [41], one case of RT-otitis media [41], and one case of optic atrophy [41]. Concern about neurocognitive toxicity has led some centres to adapt the concomitant administration of MTX during WBRT [44]. The weakness of these data lies in the fact that they are barely contextualized in the modern RT era. Unfortunately, to the best of our knowledge, there are few data regarding the more up-to-date RT techniques, with no details recorded regarding brain toxicities. Concerning toxicity, modern stereotactic radiotherapy techniques have demonstrated a significant reduction; thus, SRT is ultimately preferred as a first approach, even in multiple metastases, or suggested for histologies such as small-cell lung cancer, where WBRT has halved recurrences [64,65,66,67]. The toxicity data are scarce also for liver RT, which is mainly delivered with Cobalt-60 with a total dose of 1400–2400 rad (=14–24 Gy) to the whole liver [39,46]. No data about SRT on liver oligometastases are available.

LC was of prime importance both for BM (Table 1) and liver localization. However, considering the total dose delivered, one might wonder if GTN is a radiosensitive disease or if these response rates are due to concomitant single/multi-drug agents able to increase GTN radiosensitivity. Unfortunately, due to the absence of randomized trials and the change of the systemic regimens over the past decades, no conclusive explanations can be given.

However, from a biological point of view, the molecular features of GTN did not account for radiosensitive disease. The higher proliferation rate of GTNs can lead to a disequilibrium between pro- and antiangiogenic signals, leading to the creation of oxygen-deficient regions, which can promote RT resistance by limiting the effectiveness of RT-induced damages. Hypoxia induces radioresistance [68]; triggers the migration and invasion ability of tumours [69], also through the production of circulating tumour cells (CTCs); and induces the production of hypoxia-inducible factors (HIF), such as HIF-1, which controls the expression of genes and pathways impacting metabolism, angiogenesis, and cell growth, differentiation, survival, and death [70,71].

Bolat et al. [72] demonstrated that choriocarcinomas were associated with higher HIF1a expression levels compared to partial/complete hydatidiform moles and nonhydropic spontaneous abortions. Moreover, the same group [72] described higher levels of vascular endothelial growth factor (VEGF) and transforming growth factor (TGF)-b3 in choriocarcinomas. HIF-1a [73,74,75,76], VEGF [77,78,79], and TGF-b3 [80,81] are well-known hallmarks of radioresistance in tumour cells. In addition, it was found that in human choriocarcinoma cells, namely, BeWo b30 (a trophoblast model), oxyquinoline promoted a core form of hypoxia response gene expression as well as PDK1, NOS3, and BNIP3 gene expression and decreased PPARGC1B gene expression [82]. It should be recalled that hypoxia-induced autophagy is a significant contributory factor in antitumour treatment resistance, including with respect to RT [83]. Moreover, tumour hypoxia can result in tumour vascular mimicry and cell migration invasion caused by VEGF-A [84].

**Figure 1:**
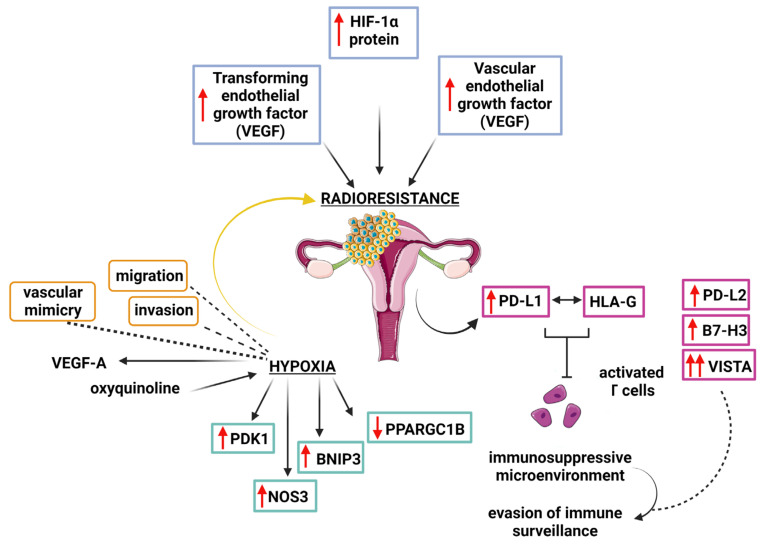
Molecular features of GTN’s radiosensitivity.

Figure 1 [85] summarises the pathways of radioresistance of GTNs described in the literature.

In recent decades, several techniques have been proposed to overcome hypoxia-induced radioresistance, including the use of radiosensitizing drugs, delivering a boost of RT dosage to hypoxic areas, and the use of high-linear-energy-transfer (LET) heavy ions [68]. High LET RT, such as ions-RT, compared to traditional photon-beam-based RT, acts independently of tumour oxygenation status [15,86]. In addition, at high LET has been described an increase in the amount of molecular oxygen that can interact with DNA and damage it, resulting in a localized oxygenated radiation response [87].

In this context, testing the potential advantages of high-LET RT would be intriguing. Moreover, high-LET RT, notably carbon ion RT, is related to a lower risk of lymphopenia, which is not negligible considering the frequent need to combine chemotherapies in metastatic settings [88].

Moreover, the biological hallmarks of GTNs and their capability to elude the immune system are fascinating. The expression of programmed cell death ligand 1 (PD-L1) was described in both placental trophoblasts and GTN, and this protein could play a role in maternal immune tolerance, on the one hand, and in tumour immune evasion on the other. Veras et al. [89] found that normal and neoplastic syncytiotrophoblasts express a high level of PD-L1 that collaborates with human leukocyte antigen-G (HLA-G) to inhibit the function of activated T cells, giving rise to an immunosuppressive microenvironment that leads to the evasion of immune surveillance. Among 112 GTNs (68 choriocarcinomas, 22 PSTTs, and 11 ETTs), Zong et al. [90] reported high levels of PD-L1, PD-L2, and B7-H3 in all samples, and the highest expression of VISTA (V-domain immunoglobulin suppressor of T cell activation), all of which are important component of immunosuppression in the tumour microenvironment. The expression of the above-reported proteins would be intriguing for the application of immunotherapy against GTNs. Moreover, from an RT point of view, it is well known that the evasion of immune surveillance is a key point of radioresistance [91] and might be overcome, in the current new era of RT, by using more modern RT techniques as well as via a combined approach with immunotherapy [92]. In this scenario, recent evidence indicates that high-LET RT can enhance immune responses to tumours and reduce their metastasizing tendency [15]. Additionally, the possibility of reducing the RT doses delivered to the surrounding normal tissues irradiated, as is executed in modern photon beam RT as well as in high-LET RT, can help to reduce the damage to circulating lymphocytes, leading to a more effective immune response [92]. Furthermore, considering the long prognoses for these patients, every effort should be made to reduce late post-actinic sequelae using highly shaped photon beam RT or high LET energies. The improvement of the management of this uncommon disease needs to be based on a better comprehension of the corresponding biological background to investigate the indications of new drugs (i.e., target therapy and immunotherapy) and their possible combination with RT in selected cases. Considering the biological background and the lessons learnt from the literature, further studies involving modern RT are warranted in cases of metastatic diseases untreatable with chemotherapy or for chemotherapy-resistant diseases. Considering the rarity of this tumour and the complexity of the management of metastatic GTNs, the treatment strategy for GTN should be individualized by a dedicated multidisciplinary team and centralized in tertiary institutions that take part in international rare tumour networks. Furthermore, the reporting of recent cases of RT for GTN delivered with the most modern techniques should be encouraged. In fact, to provide future research lines and treatment recommendations for rare diseases, even small cohorts or case reports might be of the utmost importance.

## 5. Conclusions

The clinical data concerning the use of RT for GTNs are mainly based on mono-institutional, small-cohort case series and single-case reports. Current technical developments might ensure that safe treatments can also potentially be integrated with immunotherapy, but to date, there is a lack of data. The rarity of this disease and the non-routine use of RT in metastatic settings make it difficult to plan prospective randomized trials, which, so far, have not been published. For a complete multidisciplinary approach, national and international RT registers on GTNs are encouraged in order to uncover evidence and make a step forward in the comprehension of the management of this challenging disease.

## Data Availability

Data will be shared upon reasonable request made to the corresponding author.
